# Deciphering cysteamine’s impact on cashmere-producing traits of cashmere goats: a transcriptomic and metabolomic perspective

**DOI:** 10.3389/fvets.2026.1925080

**Published:** 2026-09-17

**Authors:** Tiecheng Wu, Mengyu Guo, Tao Wang, Junyang Liu, Sarengaowa Aierqing, Leqi Yi, Bin Liu

**Affiliations:** 1 Inner Mongolia Academy of Agricultural and Animal Husbandry Sciences, Hohhot, China; 2 Alxa League Animal Husbandry and Veterinary Technology Extension Center, Inner Mongolia, China

**Keywords:** cashmere goat, cashmere-producing traits, cysteamine, metabolomics, transcriptomics

## Abstract

**Introduction:**

This study addresses the practical production problem of inferior cashmere quality in cashmere goats. We supplemented cysteamine (CS) in diets to regulate cashmere‑production‑related traits and explore its underlying nutritional regulatory pathways, so as to support the scientific and rational application of cysteamine in cashmere goat production.

**Methods:**

Sixty healthy 18‑month‑old female Inner Mongolia cashmere goats were selected for this single‑factor randomized trial and randomly allocated into three groups (*n* = 20 per group). The control group (CON) received basal diet; treatment groups were orally administrated cysteamine at 60 mg/kg body weight (BW)(LCS group) and 120 mg/kg BW (HCS group). Cashmere samples were collected from the designated trunk region. Fiber traits including diameter, staple length, breaking strength and elongation at break were measured, and fiber surface microstructure was observed by scanning electron microscopy (SEM). Skin tissues (n = 6 per group) were harvested for transcriptome and untargeted metabolomic sequencing to identify differentially expressed genes (DEGs) and differential metabolites (DEMs). KEGG enrichment and multi‑omics integrative analysis were further performed.

**Results:**

Dietary CS numerically improved cashmere yield, weight and scale height without statistical significance. The LCS treatment significantly increased straightened length, while staple length slightly decreased in the HCS group but remained higher than in CON. Initial cashmere diameter, strength and elongation at break showed no inter‑group differences; these three indicators increased after CS supplementation compared with baseline values. In total, 426 DEGs and 72 DEMs were detected, indicating obvious transcriptional and metabolic changes induced by CS. KEGG analysis highlighted divergence in the cAMP signaling pathway and thyroid hormone synthesis. DEMs were mainly enriched in lipid metabolism, organic acid metabolism and heterocyclic compound metabolism; L‑cysteine‑glutathione disulfide and α‑lipoic acid were key sulfur‑containing differential metabolites. Multi‑omics integration confirmed differences in lipid metabolism and cyclic nucleotide signaling pathways, with lipid‑related genes (*PLA2G2C, VDAC3, CHRM5, SYT4*) and cyclic‑nucleotide‑related gene IL24 significantly up‑regulated in CS groups.

**Discussion:**

Multi‑omics results indicate that cysteamine may improve cashmere‑goat productive traits by providing endogenous organic sulfur sources, accumulating sulfur‑containing metabolic intermediates and coordinating hair‑follicle‑development‑related signaling pathways. This work supplies experimental evidence for cashmere fiber quality improvement.

## 1 Introduction

Inner Mongolia Cashmere goat is a renowned native cashmere goat breed in China. It is characterized by superior cashmere quality and a distinct advantage in cashmere production relative to other cashmere breeds (1, 2). China is the world’s largest producer of cashmere (3), accounting for 50% of global output, with approximately 30% derived from Inner Mongolian cashmere goats (4). As a globally renowned genetic resource, they are prized for yielding high-quality cashmere fibers.

Cashmere fiber yield, average fiber diameter, and fiber length are crucial traits, as they determine the economic returns of cashmere production (5). Nutrition is also among the main modifiable aspects that are directly limiting the productivity of the cashmere industry and the quality of its fibers. It is hence important to ensure that efficient feed supplements that can enhance the performance of cashmere production are identified due to the nutrition control.

Keratin is the most important constituent of cashmere fibers, and it has an extremely high level of sulfur-containing amino acids (SAAs). The sulfur content directly affects the properties of cashmere fibers, including cashmere fibers (6). As an essential macro-mineral for ruminants, sulfur is critical for keratin synthesis. Studies have shown that dietary sulfur supplementation can increase the SAA content in cashmere and promote cashmere growth (7, 8). CS, an organic sulfur compound, holds application potential in this field. Previous research has reported that dietary CS supplementation increases cashmere staple length, strength, and elongation rate in cashmere goats (9). Beyond serving as an organic sulfur source, CS has been confirmed to regulate the growth hormone-insulin-like growth factor-1 (GH-IGF-1) axis, thereby influencing gene expression and metabolic processes (10, 11). Although it has been demonstrated that CS has a role in the creation of cashmere, the mechanisms behind the accumulation of metabolites and the patterns of gene expression in the goat skin hair follicles that are associated with such a growth response remain poorly studied.

In recent years, omics technologies have given a little new information regarding the biological nature of skin and how it relates to fiber properties. For instance, metabolomic analysis of skin tissues from Liaoning cashmere goats with coarse versus fine fibers identified glycylphenylalanine and taurodeoxycholate as potential key metabolites regulating cashmere growth, development, and fineness (12). Additionally, studies on mice exposed to cadmium chloride have shown widespread reduction in skin metabolites and exacerbated metabolic disorders, which are associated with aging and impaired hair regeneration (13). In genomic research on cashmere fiber diameter, most scholars have adopted strategies such as genome-wide association study (GWAS) (14) and candidate gene polymorphism analysis (15). Integrating metabolomic and transcriptomic findings enables more robust validation and reveals deeper biological mechanisms (16). Nevertheless, the overall effects of CS on the transcriptomic and metabolomic signatures and their regulation networks in the skin tissue of cashmere goats have not been elucidated.

This paper will fill the void in this area of knowledge. To find out the impact of CS on cashmere production performance, we studied the influence of various levels of CS in the diet of cashmere goats. Together with the transcriptomic and metabolomic information that was acquired with the help of skin tissue analysis, we identified the changes in gene expression profiles, metabolic fingerprints and probable regulatory networks caused by CS. It is a very important research to comprehend the molecular mechanism of regulation of CS traits of cashmere production and gives a very useful theoretical framework to the use of CS in the combined enhancement of cashmere yield and quality.

## 2 Materials and methods

### 2.1 Study design

This experiment was carried out at the Alxa White Cashmere Goat Breeding Farm located in the Inner Mongolia Autonomous Region of China. All the procedures included in the study have been reviewed and approved by the Animal Welfare and Ethical Committee of the Inner Mongolia Academy of Agriculture and Animal Husbandry Sciences (Inner Mongolia, China, No. 2026-0002). The 60 18-month-old Inner Mongolia cashmere goat ewes with identical genetic backgrounds (half-siblings), similar environmental conditions and initial body weights were chosen as the test animals. Randomized trial design in univariate form was used and the goats were divided into three groups (*n* = 20) at random. Control group was given the basal diet and the experimental groups were administered with 60 and 120 mg/kg BW^−1^ CS hydrochloride, respectively, (The included formulation contained 30 percent cysteamine hydrochloride, which is the commercially available conventional formulation widely used in ruminant feeding practice in China; hence, the amounts of the commercial formulation applied were 200 and 400 mg/kg BW^−1^ to provide the desired doses). The total time of the trial was 90 days (active growth stage (anagen) of secondary hair follicles), 10 days of the pre-trial and 80 days of formal period. CS has a foul smell and can be easily oxidized, therefore, it is commonly administered in the form of cysteamine hydrochloride in animal husbandry production. The supplementary dose of CS was referred to the studies of cashmere goats (17) and cattle (18). The cashmere goats were permitted to freely graze and drink and were put through supplementary feeding management during experimental period and provided with 0.25 kg/d (composition and nutritional value shown in Table 1) as a supplement to meet the demands of the goats. Following the grazing, treatments were applied. The medication was added to clear drench bottles and diluted by adding 100 mL of tap water per goat. The heads of all the goats were properly clamped and the solution was carefully administered into the mouth near the right side of the lips. As long as it took time to ingest all the solution, the animal was free to move. The dose and intervals of CS administration were determined as per earlier research. In the experimental period, the supplemented feed to the cashmere goats was 8.3 MJ/kg digestible energy, 14.1% crude protein, and 0.9 percent calcium; the grazing forage was 7.8 MJ/kg digestible energy, 6.5% crude protein, and 0.7% calcium. These two components formed the foundation of nutrition offered to the experimental animals, which comprised of energy and proteins. The coated CS hydrochloride that contained 30% CS hydrochloride with starch and dextrin as stabilizers was purchased by Qingdao Runbot Biotechnology (Qingdao, China). The main vegetation of this region comprised *Potaninia mongolica* Maxim., *Salsola passerina* Bunge, *Sarcozygium xanthoxylon* Bunge, *Reaumuria songarica* (Pall.) Maxim., *Ammopiptanthus mongolicus* (Maxim. ex Kom.) Cheng f., *Haloxylon ammodendron* (C. A. Mey.) Bunge, *Caragana Korshinskii* Kom, etc.

**Table 1:** Ingredient and nutrient levels of the diets fed to cashmere goats (dry matter basis).

Items	Content (%)
Ingredients	
Corn	22
Wheat bran	6
DDGS	10
Soybean meal	8
Sunflower head	9
Corn bran	20
Corn germ meal	18
Bentonite	2
Premix	5
Nutrition levels	
Metabolic energy (MJ/kg)	8.3
Crude protein (%)	14.1
Neutral detergent fiber (%)	30.6
Acid detergent fiber (%)	12.9
Calcium (%)	0.9
Total phosphorus (%)	0.4
Ash (%)	8.2

### 2.2 Animals

Sixty healthy 18-month-old Inner Mongolia white cashmere goat primiparous ewes with good body condition and consistent genetic background were selected. Their initial body weights were measured uniformly (30.53 ± 2.05 kg, *p* = 0.243), and statistical analysis confirmed no significant differences among groups. All experimental goats were numbered and equally divided into three groups (20 individuals per group) using the random number table method via completely random grouping design. During grouping, half-sib individuals from the same sire were evenly distributed into different treatment groups to balance genetic composition and eliminate confounding effects derived from family genetic differences. All goats were raised under identical feeding management, dietary formula, feeding regime and housing conditions to minimize non-experimental errors.

### 2.3 Cashmere sampling

At the end of the pre-test period, the cashmere in the 10 cm × 10 cm area of the right body side of the test goats (10 cm from the posterior edge of the scapula, slightly above the midline of the body side, Figures 1A,B for the sampling region) were removed. After the end of the test period, the cashmere samples were collected according to the area where the cashmere was removed after the end of the pre-test period, and the cashmere samples were further measured for the diameter, straight length, strength and elongation of the cashmere, and the scanning electron microscope pictures of the cashmere fibers were taken. Cashmere fiber traits (diameter, straight length, strength and elongation) were measured following the Inner Mongolia local standard *Determination Method for Straight Length of Cashmere Fiber.

**Figure 1:**
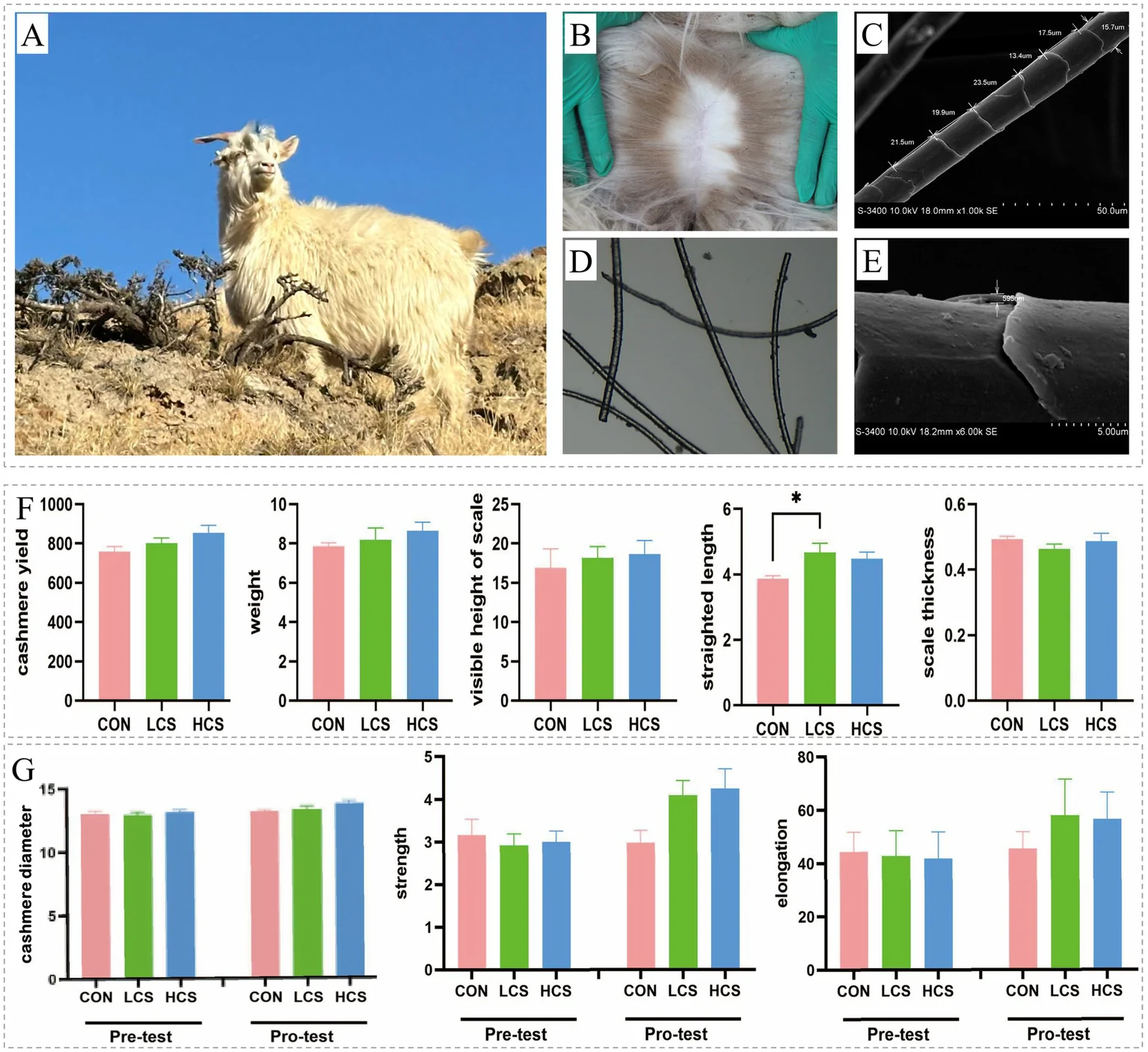
Effects of CS on cashmere performance in cashmere goats **(A,B)**. Effect of cysteamine on scale structure of cashmere **(C–E)**. Effect of cysteamine on performance traits of cashmere **(F,G)**. *Indicates significant difference between groups at *p* < 0.05 level. The control cysteamine group is shown in pink, the low-dose cysteamine group is shown in green, the high-dose cysteamine group is shown in blue.

Fiber diameter was determined with an OFDA 2000 fiber fineness analyzer, strength and elongation with a YG001A electronic single-fiber strength tester. SU8100 scanning electron microscope was used to observe and measure fiber diameter, scale thickness, and scale morphology, with micrographs collected.

For each individual, 100 fibers were randomly selected for measurement, and the average was used as the final phenotypic data.

### 2.4 Determination of cashmere performance indexes

#### 2.4.1 Cashmere diameter

Cashmere samples were separated from the mixture containing coarse cashmere and cashmere fiber, and the cashmere was washed four times with carbon tetrachloride in order to remove the oil and dirt on the cashmere surface. The diameter of cashmere fiber was determined using OFDA 2000 (OFDA, Australia).

#### 2.4.2 Straightened length

Gently straighten a single cashmere fiber with forceps, measure the length of the cashmere fiber in the straightened state, and measure 100 pile fibers for each sample, and the measurement results were averaged.

#### 2.4.3 Strength and elongation

YG001A electronic single fiber strength machine (Wenzhou, China) was used to measure the strength and elongation of cashmere. The tensile speed was 10 mm/min, the clamping distance was 10 mm, the starting force was 5 cN, and the pre tension was 3 cN. The instrument automatically records the maximum strength, tensile length, elongation and other information when the fiber breaks. Ten cashmere fibers were measured for each sample, and the measurement results were averaged.

#### 2.4.4 Scanning electron microscope

The cashmere samples (middle section of cashmere fiber) of 3 experimental goats with similar physical condition and consistent hair follicle development status were randomly selected for each group, and 10 SEM images were randomly taken for each sample. The samples were degreased in carbon tetrachloride solution, and then the degreased cashmere samples were successively put into 30, 50, 70, 80, 90, 95, 100% alcohol for 15 min each time, and isoamyl acetate for 15 min. The samples were put into the K 850 critical point dryer (Quorum, UK) for drying, and then the samples were tightly attached to the conductive carbon film double-sided adhesive tape and placed on the sample table of the MSP-2S ion sputtering instrument (IXRF, America) for gold spraying for about 30 s. The fiber diameter, scale thickness, scale type and morphology were observed and measured under the SU 8100 scanning electron microscope (Hitachi, Japan). All image observations and index determinations were performed in a blind manner to avoid subjective evaluation bias. Individual experimental goats were regarded as statistical units in this study, and the average values measured from multiple images of the same sample were adopted for subsequent data analysis.

#### 2.4.5 Cashmere yield

Cashmere yield refers to the weight of raw cashmere mixture (containing coarse cashmere and natural impurities) artificially harvested from cashmere goats during the telogen phase of secondary hair follicles.

#### 2.4.6 Skin sampling

At the end of the experiment, six goats in each group were randomly selected, and the skin was prepared for surgery in the villus collection area. After disinfection, the skin was drilled to the subcutaneous tissue with a skin sampler, the scalpel was stripped along the upper edge of the subcutaneous tissue, the residual blood was rinsed with RNase free water, the surface water was sucked dry, quickly put into a cryopreservation tube and placed in liquid nitrogen, and stored at −80 °C in the laboratory. The wounds of experimental goats were disinfected, hemostatic and bandaged. Four pieces of skin tissue were collected from each goat for transcriptome, metabolome and subsequent assays. All sampling instruments were disinfected.

### 2.5 RNA isolation, cDNA library construction, sequencing and data analysis

A total of 18 cashmere goat skin tissues were collected from three treatment groups (*n* = 6) for transcriptome sequencing. Total RNA was extracted using a TIANGEN Polysaccharide Polyphenol Kit (Qiagen, Germany), and then using the kit NEBNext Ultra™ Directional RNA Library ep Kit for Illumina. RNA integrity and concentration were assessed using the RNA Nano 6000 Assay Kit of the Bioanalyzer 2100 system (Agilent Technologies, CA, USA). The mRNA was purified from total RNA using poly-T oligo-attached magnetic beads, and randomly interrupts mRNA. Using fragmented mRNA as template and random oligonucleotides as primers, cDNA was synthesized under M-MuLV Reverse Transcriptase and DNA polymerase I system. The cDNA was end repaired, a-tailed and ligated with sequencing adapters. The cDNA of 370–420 bp was screened with AMPure XP system (Beckman Coulter, Beverly, USA), amplified by PCR and purified, and finally the library was obtained. Dilute the library to 1.5 ng/ul. Sequencing was performed using an Illumina NovaSeq 6000 paired-end sequencing system (Illumina, USA). The clean data were obtained by removing reads containing adapter, N base and low quality from raw data. The clean reads were aligned with the goat reference genome (ARS1) using Hisat2 v2.0.5 to obtain the location information of reads on the reference genome (19). According to the location information, the Feature Counts v1.5.0-p3 was used to count the reads numbers mapped to each gene (20). And then FPKM (fragments per kilobase of transcription per million mapped reads) of each gene was calculated based on the length of the gene and reads count mapped to this gene (21). The library construction and sequencing were performed by Beijing Novo gene Technology Co., LTD (China).

### 2.6 Identification and enrichment analysis of DEGs

DESeq2 software was used to perform differential expression analysis between the three comparison combinations. FDR < 0.05 and |log2(foldchange)| > 1 were set as the threshold for significantly differential expression. Go (Gene Ontology) enrichment of DEGs and KEGG (Kyoto Encyclopedia of Genes and Genomes) pathway enrichment analysis were performed by cluster Profiler (3.8.1).

### 2.7 Quantitative real-time polymerase chain reaction verification

Reverse transcriptase quantitative polymerase chain reaction (qRT-PCR) was used to verify the RNA-seq analysis of randomly chosen 10 DEGs. The reverse transcription was performed using Evo M-MLV RT Mix Kit (Accurate Biotechnology, China). All RNA samples were reverse transcribed using 5x Evo m-mlv RT reaction mix reverse transcription reagent. The reaction conditions were: 37 °C for 15 min, 85 °C for 15 s, and 4 °C for storage. The qRT-PCR was carried out using a ChamQ Universal SYBR qPCR Master Mix (Vazyme, China). Bimake 2x SYBR Green qPCR Master Mix fluorescence Quantification Kit was used for fluorescence quantification of mRNA reverse transcripts. The reaction conditions were: pre denaturation at 95 °C for 3 min; Denaturation at 95 °C for 10 s, annealing at 60 °C and extension for 30 s, and then enter the dissolution curve program after 40 cycles; 95 °C for 5 s, 60 °C for 30 s. GAPDH was used as an internal control. The 2^−△△Ct^ method was used to determine the relative abundance of transcripts. For accuracy, the whole experiment was conducted thrice. All primers used in this study are listed in Table 2.

**Table 2:** Primer sequences of real-time.

Gene name	Login number	Primer sequences (5′ → 3′)	Product length/bp
AGT	XM_005699373.3	F: GAGAAGTTCTTGGCGGTGGTGAG	130
		R: CCATCTTCCGATTCCAGGCACAG	
AOC1	XM_005679653.3	F: GTGTAAGGAATCTGAGGCTGATGGC	124
		R: AGGTAGAGGAGGCGGCAGTTTC	
COL28A1	XM_005678961.3	F: TTACCTGGAGTTGGAGACACTGGAG	117
		R: TTTCTGCCCGATGTCACCCTTTG	
CXCL10	NM_001285721.1	F: ACCAAGCCAGTCACCAAATCAGC	118
		R: GCCTCGGTATAGCTTACAGTCACAG	
CYP1A1	XM_005695137.3	F: TGATGGCACCGTCAACAAAG	101
		R: CTTCTGCTGCCTTCCAGATCA	
HHATL	XM_005695586.3	F: CAATGTCATCATCTTCGCCCTCTCC	150
		R: AGCAGCAGCAGCAAGTACCAAG	
IL12RB2	XM_018045355.1	F: GATCTCCGTCGGTGTTGTTCCAG	107
		R: GGTGGGTGTCTCGTCCTCTGTC	
KRTAP15-1	NM_001285771.1	F: TGGCCTTGGGTCTTTTGGTT	107
		R: AAGATGACCGGCAACTCCTG	
PLA2G2C	XM_005676905.3	F: CCGTGGTCTGTGAATGTTCCCTTG	106
		R: GGCAGGTTCTCTTTGAAGCAGTAGG	
GAPDH	XM_005680968.3	F: CCACGAGAAGTA TAACAACACC	120
		R: GTCA TAAGTCCCTCCACGAT	

### 2.8 LC–MS (liquid chromatograph mass spectrometer) for metabolite determination and analysis

Each sample of skin tissue (100 mg) was separately ground with liquid nitrogen and then the homogenate was resuspended in a pre-chilled solution of 80% methanol and 0.1% formic acid by vigorous vortexing. The samples were put on ice followed by incubation on it (5 min) and afterwards centrifuged at 15,000 g, 4 °C, 20 min. Others supernatants were diluted to end concentrations with 53% methanol and water of LC–MS purity. The samples were subsequently poured into a new Eppendorf tube and re-centrifuged at 15,000 g, 4 °C, 20 min. As the last step, the supernatant was entered into the LC–MS/MS system analysis.

UHPLC–MS/MS analysis was performed on a Vanquish UHPLC system (ThermoFisher, Germany) and an Orbitrap Q ExactiveTMHF-X mass spectrometer (Thermo Fisher, Germany) at Novogene Co., Ltd. (Beijing, China). They were eluted from a Hypesil Gold column (100 × 2.1 mm, 1.9 um) with a flow rate of 0.2 mL/min, with a 17-min linear gradient. Positive polarity mode: eluents: eluent A (0.1% FA in Water) and eluent B (Methanol); negative polarity mode: eluent A (5 mM ammonium acetate, pH 9.0) and eluent B (Methanol). The program of the solvent gradient was 2% B, 1.5 min; 2 to 85% B, 3 min; 85 to 100% B, 10 min; 100 to 2% B, 10.1 min; and 2% B, 12 min. The Q ExactiveTM HF-X mass spectrometer was used in positive/negative polarity mode, spray voltage 3.5 kV, capillary temperature 320 °C, sheath gas flow rate 35 psi, and aux gas flow rate 10 L/min, S-lens RF 60, Aux gas heater temperature 350 °C.

The raw data files were processed in Compound Discoverer 3.1 (CD3.1, ThermoFisher) to do peak alignment, peak picking and quantification of each of the metabolites. The following settings were used as main parameters: tolerance of retention time, 0.2 min; tolerance of real mass, 5 ppm; tolerance of signal intensity, 30%; signal-to-noise ratio, 3; and minimal intensity. Peak intensities were then normalized to the total spectral intensity. Normalized data showed the molecular formulae depending on the additive ions, molecular ion peaks, and fragment ions. Later on, peaks were compared with the mzCloud, mzVault and MassList databases to get precise qualitative and comparative quantitative results.

### 2.9 Metabolite data analysis to obtain differential metabolites

These metabolites were annotated using the KEGG database, HMDB database and LIPID Maps database. Principal components analysis (PCA) and Partial least squares discriminant analysis (OPLS-DA) were performed at metaX. After FDR multiple testing correction, the metabolites with VIP > 1 and *p* < 0.05 was considered to be differential metabolites. Volcano plots were used to filter metabolites of interest which based on log2 (Fold Change) and −log10 (*p*-value) of metabolites. For clustering heat maps, the data were normalized using *z*-scores of the intensity areas of differential metabolites. The functions of these metabolites and metabolic pathways were studied using the KEGG database.

### 2.10 Joint analysis of metabolites and genes

Metabolites and genes in the same pathway share a potential association in dysregulation, so we utilized a pathway-based approach and integrated different levels of omics in the biological process. Enriched differential genes and metabolites were used in the joint pathway module for integrative analysis in MetaboAnalyst5.0. After uploaded our differential metabolites on MetaboAnalyst,^1^ the metabolites were then mapped to KEGG metabolic pathways for enrichment analysis. Correlation analysis based on Pearson^2^ correlation coefficients was performed between significantly DEGs identified via transcriptomic analysis and significantly DEMs screened from metabolomic profiling, so as to quantify the association strength between DEGs and DEMs. A Pearson correlation coefficient below zero indicates a negative correlation, whereas a value above zero represents a positive correlation. The top 50 differential metabolites (ranked by ascending *p*-value) and top 100 differentially expressed genes (ranked by ascending *p*-value) were visualized. If the total number of differential metabolites was less than 50 or the total number of DEGs was less than 100, all identified differential metabolites or DEGs were retained for presentation, respectively. Network diagrams enable intuitive visualization of the associations between metabolites and genes. The top 10 screened differentially expressed genes and top 5 differential metabolites were selected for network construction. In the graph, metabolites are marked in yellow and genes in blue, with straight lines representing pairwise correlations between them.

### 2.11 Statistical analysis of data

Cashmere performance data are expressed as the mean and standard error of the mean (SEM). Comparisons were made by one-way analysis of variance (ANOVA) and Duncan’s multiple-range test using SPSS (version 19.0, IBM SPSS, Chicago, IL, USA). All measured variables satisfied the normal distribution and homogeneity of variance assumptions for one-way ANOVA. Statistical difference was, respectively declared as significant or highly significant at *p* < 0.05 or *p* < 0.01, while trend was discussed at 0.05 < *p* ≤ 0.10.

## 3 Results

### 3.1 Cashmere performance in response to CS supplementation

Dietary CS supplementation induced a increase in cashmere yield, fleece weight and scale height, although the differences did not reach statistical significance. The LCS treatment significantly elevated straightened length; straightened length was slightly lower in the HCS group but remained greater than that of the control group. No obvious alterations were observed in scale thickness across all treatments (Figure 1F).

Before the experiment, the values of cashmere diameter, strength, and elongation were relatively close among the three groups. After the experiment, all three parameters were increased to varying degrees in the three groups, with small intergroup differences (Figure 1G).

### 3.2 Skin transcriptomics profiles in response to CS supplementation transcriptome sequencing and gene expression analysis

RNA-seq was used to study transcriptome changes in skin tissue of test goats during CS supplementation. Totals of 832,934,928 raw reads were generated from 18 samples; after filtering, 815,606,370 high-quality clean reads were obtained. The values of Q30 of clean reads ranged from 89.58 to 94.99%, and the contents of G and C bases accounted for about 51.57% of the total number of bases. Comparing clean reads with the reference genome (ARS1), the mapped ratio comparison rate of samples exceeded 93.88%, indicating that the quality of RNA-Seq data was sufficient and could be used for subsequent analysis (Table 3). Based on the information from clean reads were aligned to reference genome, filter reads that are aligned to multiple positions and gene overlap regions in the genome. The threshold for gene expression is FPKM greater than 1. 15,429 genes were identified in the CON group, 15,450 genes were identified in the LCS group, and 15,590 genes were identified in the HCS group. The unique expression genes of CON group, LCS group, and HCS group were 195, 191, and 278, respectively. The number of shared expressed genes among the three groups accounts for 92.68% of the total expressed genes (Supplementary Figure S1), indicating that there are a large number of co expressed genes in the skin tissue of cashmere goats between different treatments.

**Table 3:** Transcriptome sequencing data of all samples.

Samples	Raw reads	Clean reads	Clean ratio	Q30	GC_pct	Total mapped reads	Mapped ratio
CON_1	46,069,390	44,104,640	95.74	93.82	51.79	42,501,155	96.36
CON_2	47,535,768	46,851,154	98.56	94.88	51.87	45,440,431	96.99
CON_3	45,256,032	43,785,846	96.75	94.31	52.72	42,386,549	96.8
CON_4	45,366,256	44,643,528	98.41	92.64	51.69	42,752,057	95.76
CON_5	41,360,760	40,843,240	98.75	91.22	51.29	39,195,403	95.97
CON_6	46,164,512	44,565,146	96.54	91.42	50.83	42,963,423	96.41
LCS_1	46,040,426	45,216,780	98.21	92.52	52.13	43,833,459	96.94
LCS_2	49,605,702	48,938,422	98.65	94.99	51.28	47,434,443	96.93
LCS_3	50,129,974	49,112,638	97.97	90.97	49.88	47,421,027	96.56
LCS_4	44,523,740	43,921,412	98.65	93.03	52.15	42,177,502	96.03
LCS_5	46,641,738	45,828,898	98.26	92.9	52.23	44,011,706	96.03
LCS_6	49,532,664	48,535,778	97.99	90.32	51.51	46,199,372	95.19
HCS_1	47,300,608	46,524,610	98.36	94.51	51.82	45,068,526	96.87
HCS_2	45,732,240	45,126,216	98.67	94.67	51.58	43,666,115	96.76
HCS_3	46,733,378	44,872,158	96.02	94.15	52.04	43,439,161	96.81
HCS_4	46,300,698	45,664,980	98.63	89.58	50.94	42,870,028	93.88
HCS_5	47,349,454	46,313,902	97.81	92.15	50.74	44,282,626	95.61
HCS_6	41,291,588	40,757,022	98.71	92.65	51.81	39,150,587	96.06
Average	46,274,163	45,311,465	97.93	92.82	51.57	43,599,643	96.22

### 3.3 Identification and analysis of differentially expressed genes

By supplementing CS, 193 (116 up-regulated and 77 down-regulated) and 238 (116 up-regulated and 122 down-regulated) DEGs were differentially expressed in HCS vs. CON and LCS vs. CON respectively (Supplementary Tables S2, S3). In the comparison between HCS vs. LCS, a total of 253 DEGs were identified, among which 149 were upregulated and 104 were downregulated (Figure 2A, Supplementary Table S4). No common DEGs were identified among the three groups. Specifically, there were 48 overlapping DEGs between the HCS vs. CON comparison and the HCS vs. LCS comparison, 49 overlapping DEGs between the HCS vs. LCS comparison and the LCS vs. CON comparison, and 32 overlapping DEGs between the HCS vs. CON comparison and the LCS vs. CON comparison (Figure 2B). The DEGs of all the comparison groups were merged as the differential gene set, and the FPKM values of the genes were analyzed by hierarchical clustering. A hierarchical clustering heat map was generated to illustrate the gene expression patterns in skin tissues of cashmere goats after cysteamine supplementation (Figure 2C). The gene expression patterns differed among the three groups. Compared with the CON group, the LCS group significantly increased the expression levels of 116 genes, including *GALNTL6, CDC20, PLA2G2C, IL24, CNN2*, and *ARPC1A*, while significantly decreasing the expression levels of 122 genes, such as *CDCP2, TKDP1, AGT, FFAR3, CPNE7*, and *TYRP1*.

**Figure 2:**
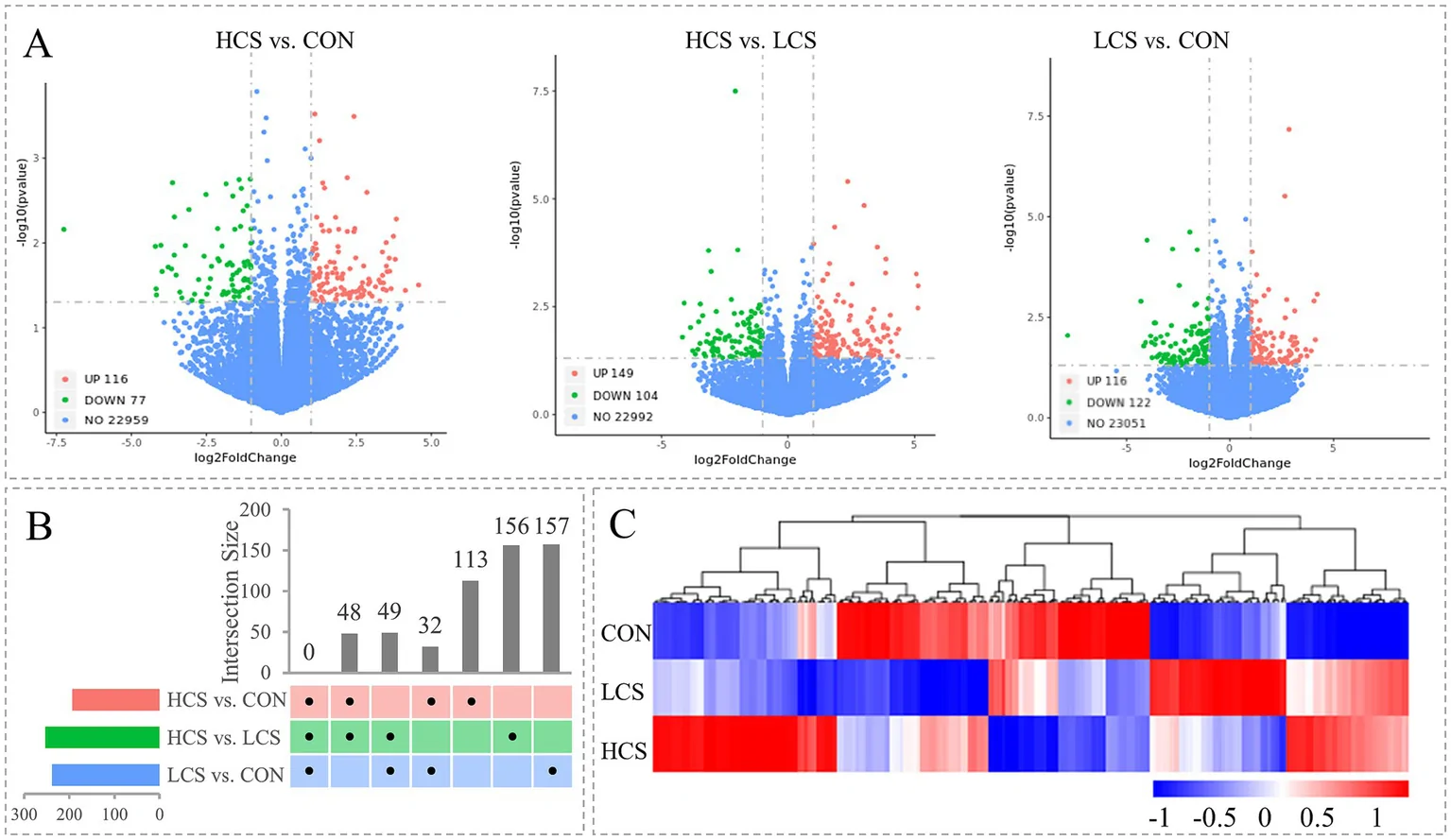
Volcanic map of DEGs **(A)**. Up-regulated DEGs are shown in red dot notation, down-regulated DEGs are shown in green dot notation. Combination chart of DEGs **(B)**. Black dots represent the differentially expressed genes detected in this group. Heatmap clustering of DEGs **(C)**. Red indicates up-regulated gene expression, and blue indicates down-regulated gene expression.

In comparison with the CON group, the HCS group significantly upregulated the expression of 116 genes (e.g., *CHRM5, SNORA55, SYT4, ROPN1L, VDAC3*, and *CBY3*) and significantly downregulated the expression of 77 genes, including *FADS2P1, MUC5B, BHMG1, GJB7, CD79B*, and *RNF183*.

### 3.4 Functional annotation analysis of DEGs

KEGG enrichment analysis revealed 7, 16, and 4 significantly enriched pathways (*p* < 0.05) in the comparisons of LCS vs. CON, HCS vs. LCS, and HCS vs. CON, respectively (Supplementary Table S5). As illustrated in Figure 3A, the top 15 significantly enriched pathways (with *p*-values ranging from 0 to 1) were analyzed for each comparison group (HCS vs. CON, HCS vs. LCS, and LCS vs. CON). For HCS vs. CON, DEGs were enriched in pathways such as thyroid hormone synthesis, steroid hormone biosynthesis, ribosome, TRP channel signaling pathway, and chemical carcinogenesis—receptor activation. For HCS vs. LCS, DEGs were highly and significantly enriched in pathways including the cyclic adenosine monophosphate (cAMP) signaling pathway, neuroactive ligand—receptor interaction, vascular smooth muscle contraction, and cell adhesion molecules.

**Figure 3:**
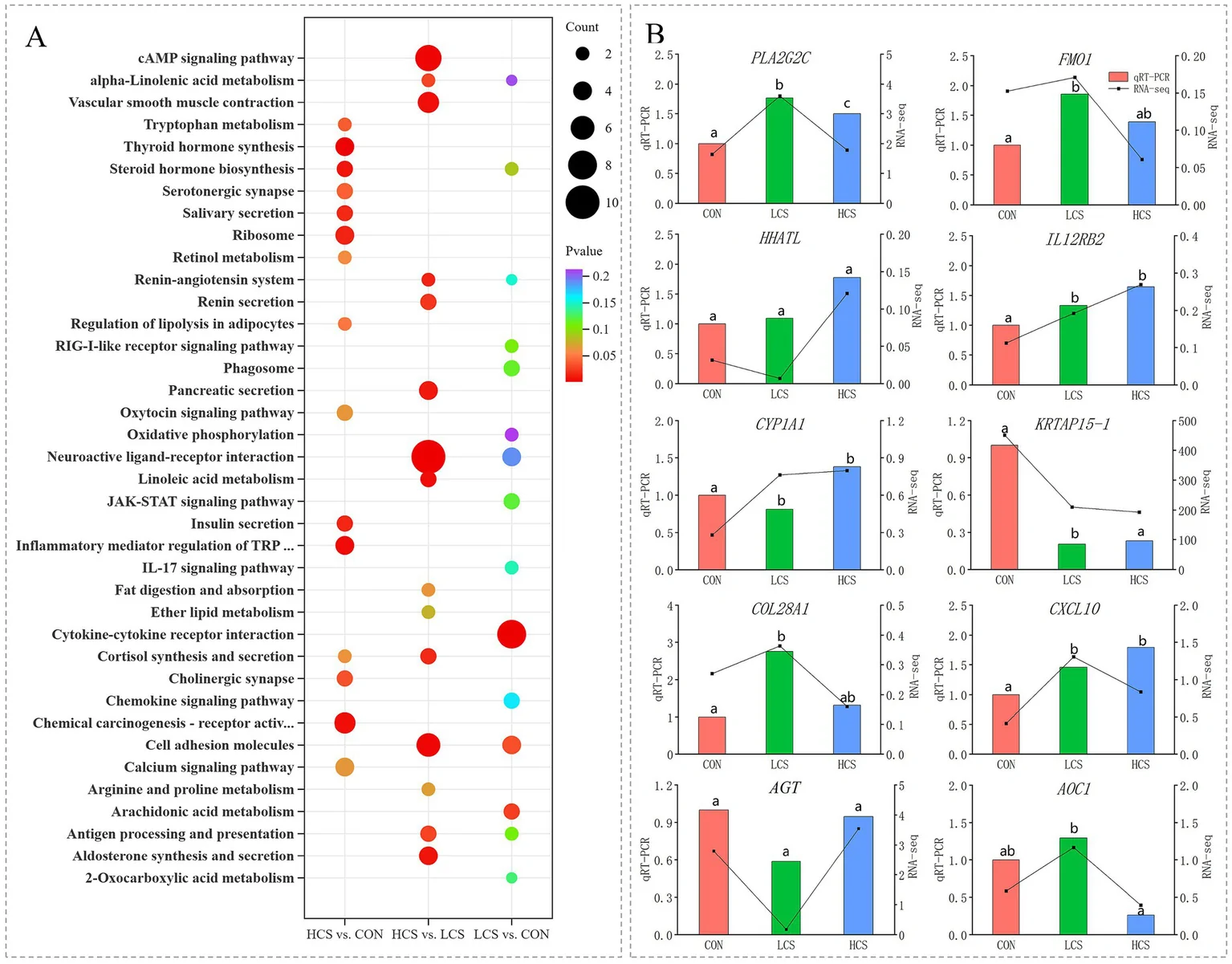
KEGG enrichment bubble chart **(A)**. The bubble size represents the number of differential molecules (genes/metabolites) in the pathway, and the closer the color is to red (the smaller the *p*-value < 0.05), the stronger the statistical significance of the pathway. Validation of RNA-seq results by qRT-PCR **(B)**. Bars sharing no common lowercase letters indicate significant differences between groups determined by Tukey’s multiple comparisons test (*p* < 0.05); bars with shared letters are not significantly different.

In contrast, DEGs in the LCS vs. CON comparison were enriched in the cytokine—cytokine receptor interaction pathway and arachidonic acid metabolism pathway. The aforementioned pathways mainly include the bile secretion pathway, tryptophan metabolism pathway, cyclic guanosine monophosphate-protein kinase G (cGMP-PKG) signaling pathway, renin secretion pathway, vitamin digestion and absorption pathway, and regulation of lipolysis in adipocytes pathway. Due to differences in CS dosage, the three groups exhibited distinct variations in the type, degree, and significance of pathway enrichment: The HCS vs. LCS group was primarily enriched in signal transduction and metabolic regulation—related pathways, which can affect cashmere yield, quality, and growth cycle by regulating the core processes of hair follicle development. The LCS vs. CON group focused on cytokine interaction and inflammatory metabolism pathways, which indirectly influence the growth stability, yield, and fiber quality of cashmere by regulating hair follicle microenvironment homeostasis, cell proliferation and differentiation, and stress adaptation. The HCS vs. CON group was more closely associated with hormone synthesis and basic metabolic pathways, which directly impact key cashmere traits by regulating growth signals and providing energy and material substrates.

### 3.5 Validation of RNA-Seq expression levels by qRT-PCR

To verify the reliability of the RNA-seq data, the mRNA abundances of 10 DEGs (*PLA2G2C*, *KRTAP15-1*, *HHATL*, *IL12RB2*, *CYP1A1*, *FMO1*, *COL28A1*, *CXCL10*, *AGT*, *AOC1*) were identified using qRT-PCR (Figure 3B). Six biological replicates were arranged in each group, and three technical replicates were performed for each sample. Furthermore, the expression stability of the reference gene GAPDH was verified prior to the qRT-PCR experiments. The 10 DEGs identified by qRT-PCR varied significantly among groups, similar to those observed by RNA-seq analysis (Table 3). Although the fold changes were not exactly as shown by the transcriptome analysis data, all the validated genes showed similar expression patterns.

### 3.6 Skin metabolomic profiles in response to CS supplementation

To further analyse the influence of CS on skin tissues of cash mere goats, we performed a non-targeted metabolomic analysis of skin tissues using UHPLC–MS/MS (Supplementary Figure S2). A total of 740 metabolites were annotated through the KEGG, HMDB and LIPID MAPS databases (Supplementary Table S6). The three groups shared the same metabolite categories, including 116 organic acids and derivatives (the super class level is the same as below), 101 lipids and lipid-like molecules, 47 organoheterocyclic compounds, 37 organic oxygen compounds, 37 Nucleosides, nucleotides and analogs, 17 benzenoids, 11 organic nitrogen compounds, 7 phenylpropanoids and polyketides,1 lignans, neolignans and related compounds and 366 unclassified metabolites. According to the OPLS-DA (Supplementary Figure S3A), metabolic profiles varied among groups. Response permutation test plots, presented in (Supplementary Figure S3B), represent the OPLS-DA model assessment parameters between the groups. The OPLS-DA models of LCS vs. CON and HCS vs. CON were characterized by *R*2 = 0.93, 0.94 and *Q*2 = −0.44, −0.57, respectively. DEMs screening conditions were VIP > 1.0 and *p* < 0.05. In the comparative analyses of HCS VS CON, HCS VS LCS, and LCS VS CON, 25, 30, and 17 unique metabolites were identified, respectively (Figure 4A). Among the differential metabolites, The largest number are lipids and lipid-like molecules (10 species) and organic acids and derivatives (10 species), followed by organoselenium compounds (7 species; Figure 4B, Supplementary Table S8). To analyze the changes of metabolites during development, we performed clustering and classification of differential metabolites (Supplementary Table S7). The metabolites were divided into six clusters: Cluster 1 showed a pattern of “first increase and then slight decrease,” with a significant increase in the LCS group and a slight decrease in the HCS group; metabolites in Cluster 2 and Cluster 5 were significantly detected in CON but had low contents in HCS, showing a pattern of “continuous strong down-regulation”; metabolites in Cluster 3 and Cluster 4 had the highest concentration in LCS and then decreased in the HCS group, showing a fluctuating pattern of “first increase and then decrease”; Cluster 6 showed a fluctuating pattern of “first decrease and then increase,” with a significant decrease in the LCS group and a recovery in the HCS group (Figure 4C).

**Figure 4:**
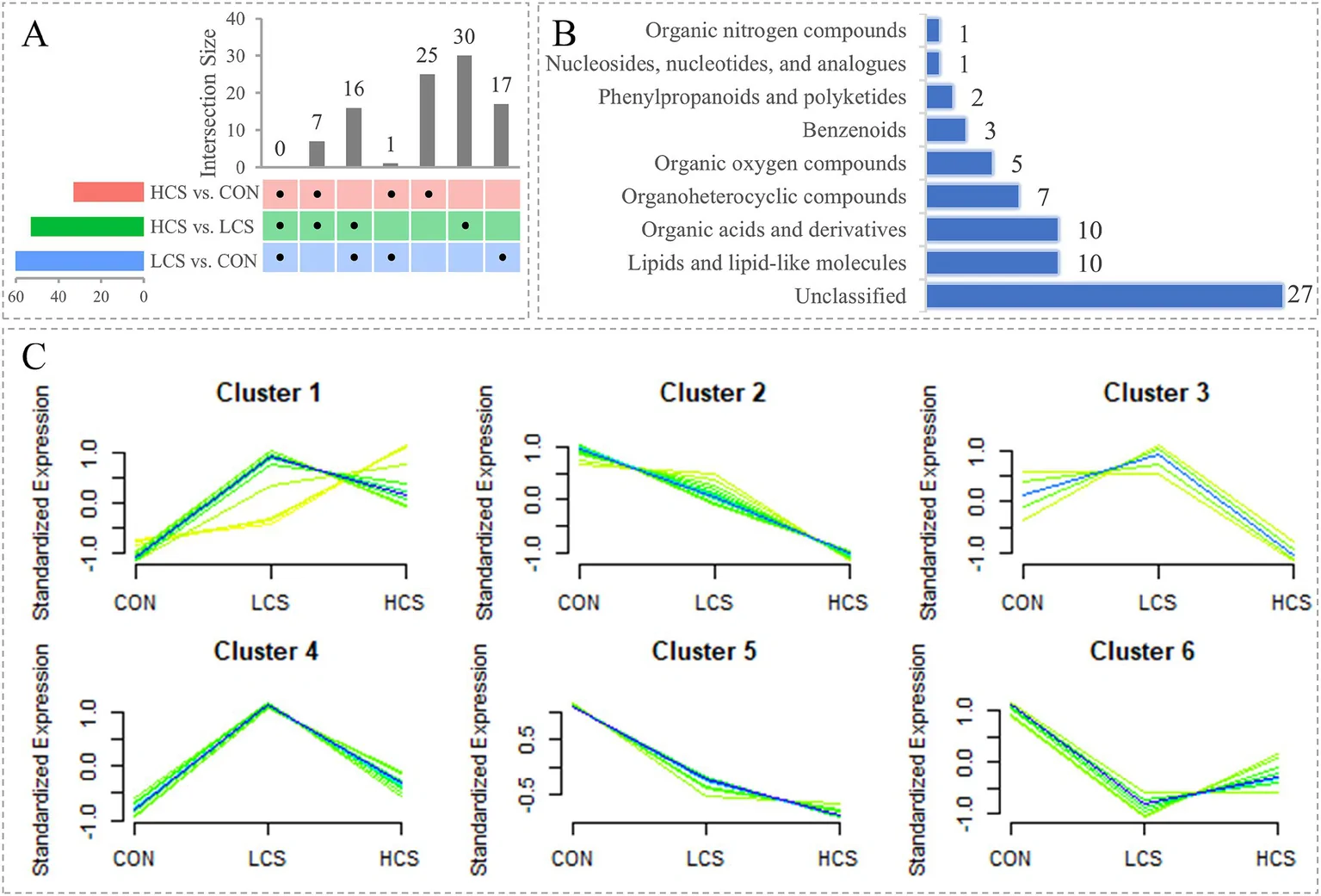
Variability characteristics of differential metabolites among samples **(A)**. Black dots represent the differentially expressed metabolites detected in this group. Classify the differential metabolites **(B)**. Cluster and classify the differential metabolites **(C)**. Clustering analysis of all differential metabolites was conducted to demonstrate their expression patterns in the three groups.

### 3.7 Association analysis of transcriptome and metabolome data

In this study, Pearson correlation analysis was applied to explore the associations between genes and metabolites in this study. The screening threshold of correlation coefficient was set as |*r*| > 0.7. A total of twelve biological samples were used for each group. Multiple testing correction was performed to reduce false positive results. All correlation analyses were conducted independently within each treatment group instead of being calculated based on all pooled samples. The obtained DEGs and DEMs were simultaneously mapped to the KEGG pathway database to explore the pathways co-involved by both. As shown in Figure 5E, the shared pathways of DEGs and DEMs between the LCS and CON groups include platelet activation, bile secretion, aldosterone synthesis and secretion, tryptophan metabolism, renin secretion, and arachidonic acid metabolism pathways. As shown in Figure 5A, the shared pathways of DEGs and DEMs between the HCS and CON groups include histidine metabolism, mTOR signaling pathway, PI3K-Akt signaling pathway, thyroid hormone synthesis, bile secretion, tryptophan metabolism, cGMP-PKG signaling pathway, cAMP signaling pathway, and renin secretion pathways. As shown in Figure 5C, the shared pathways of DEGs and DEMs between the HCS and LCS groups include tryptophan metabolism, taurine and hypotaurine metabolism, histidine metabolism, glycine, serine and threonine metabolism, and carbon metabolism pathways. Therefore, we speculate that CS, as a dietary additive, participates in the bile acid secretion, thyroid hormone synthesis, tryptophan metabolism, and renin secretion pathways in the skin of cashmere goats, which provides a certain reference for understanding the metabolic network of CS in cashmere goats. Metabolite-gene association network analysis indicated close correlative relationships between metabolites and genes, which presented relatively strong correlation patterns (Supplementary Table S1). In Figure 5B, CASR exhibited strong positive and negative correlations with most metabolites, CASR exhibits a significant positive correlation with 5′-Adenylic acid, and is significantly negatively correlated with LSD-d3. In Figure 5D, Strong positive correlations were observed between two genes (*SEZ6L*, *USP49*) and oleanolic acid, hippuric acid as well as α-lapachone. Conversely, *PGR* displayed marked negative correlations with these three metabolites, revealing opposing regulatory relationships between *PGR* and the two candidate genes in the metabolite-gene association network. In Figure 5F, Genes *SAXO2* and *ACTR5* were significantly positively correlated with 4-hydroxyphenyllactic acid and DL-4-hydroxyphenyllactic acid, while they exhibited significant negative correlations with estropipate, penicillin G, and indole-3-acetic acid. The gene-metabolite correlation network is enriched with numerous pairs exhibiting robust positive and negative correlations, uncovering bidirectionally divergent influence patterns within cashmere-associated metabolic pathways. Importantly, every pairwise relationship shown herein constitutes only statistical Pearson correlation rather than validated direct causal regulation.

**Figure 5:**
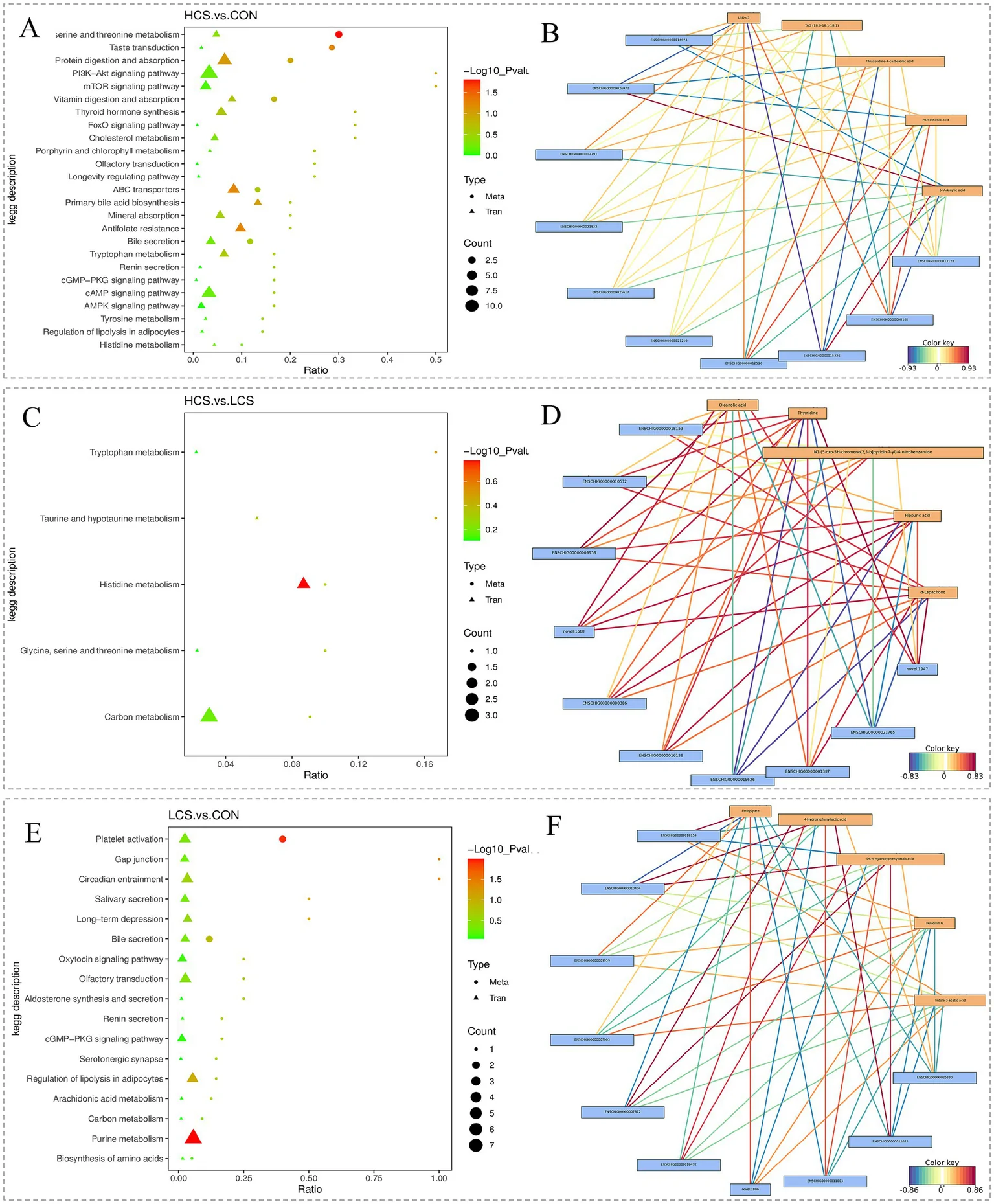
KEGG enrichment bubble chart **(A,C,E)**. Triangles represent differentially expressed genes (Tran), and dots represent differentially expressed metabolites (meta). The size of the dots/triangles corresponds to the number of differential molecules (count); the larger the size, the more differential molecules are involved in the pathway. The right panel shows the correlation network diagram **(B,D,F)**, which illustrates the associations between genes and metabolites.

## 4 Discussion

Cashmere is a keratinous fiber produced by the secondary hair follicles of cashmere-producing goats, and its growth is comprehensively regulated by multiple factors including breed, rearing environment, and dietary nutrition, among which nutritional status is considered to play a pivotal role (22, 23). Sulfur-containing amino acids are the first limiting amino acids for wool-producing sheep (24), and increased dietary intake of these amino acids has been demonstrated to promote wool and hair fiber growth in both sheep (25) and goats (26). Cashmere is primarily composed of high-sulfur keratins, and sulfur not only influences fiber yield but also critically determines quality traits such as elasticity and tensile strength, predominantly existing in the forms of cystine, cysteine, and methionine within the keratin structure. CS possesses dual characteristics as both an organic sulfur donor and an endocrine regulatory factor. It has been shown to reduce somatostatin levels, thereby alleviating the inhibitory effect on GH secretion, which indirectly upregulates the IGF-1 signaling axis and subsequently modulates digestive metabolism and hair follicle development (27, 28). Previous studies have reported that dietary supplementation with 10–100 mg/kg body weight of cysteamine can improve feed conversion efficiency (29), nutrient apparent digestibility (30), lactation performance (31), and cashmere quality (9), indicating its potential application in cashmere goat production.

The present study was designed to systematically investigate the regulatory effects and underlying molecular mechanisms of CS on cashmere growth in Inner Mongolia cashmere goats, with a particular focus on its sulfur nutritional properties and endocrine regulatory functions. Phenotypically, dietary supplementation with varying doses of CS resulted in increasing trends in cashmere yield, fiber breaking strength, and elongation rate, although no statistically significant differences were observed between treatment groups and the control group. This outcome is likely attributable to the experimental period not fully covering the complete cashmere growth cycle, which spans approximately 6 months. Notably, CS significantly increased the stretched length of cashmere fibers, while no significant alterations were observed in fiber diameter or cuticle microstructure. These findings are consistent with those reported by Liu et al. in Inner Mongolia white cashmere goats, further supporting the notion that an appropriate dosage of CS can effectively improve cashmere length traits without compromising fiber fineness or surface structural integrity.

At the transcriptomic level, RNA sequencing revealed that the LCS yielded 238 differentially expressed genes (116 upregulated and 122 downregulated), whereas the HCS yielded 193 differentially expressed genes (116 upregulated and 77 downregulated), confirming that CS can reshape the cutaneous transcriptional landscape and broadly interfere with biological processes related to hair follicle development. Among these, genes closely associated with cell proliferation and differentiation, including *KRTAP15-1*, *IL12RB2*, and *PLA2G2C*, exhibited significant differential expression, suggesting that CS may activate signaling pathways governing hair follicle stem cell proliferation and differentiation, and providing a structural foundation for cashmere growth. KEGG pathway enrichment analysis further demonstrated that the differentially expressed genes were significantly enriched in pathways including bile secretion, the cAMP signaling pathway, thyroid hormone synthesis, and steroid hormone biosynthesis. These pathways are intimately associated with hair follicle morphogenesis and cyclic development: bile acid metabolism, mediated by core receptors such as FXR and TGR5, regulates cutaneous lipid homeostasis, energy metabolism, and immune microenvironment, thereby establishing a favorable milieu for follicle growth (32); cAMP signaling and thyroid hormone synthesis are directly involved in follicle morphogenesis and functional maintenance (33, 34), critically determining cashmere growth efficiency and quality formation; and steroid hormone biosynthesis may indirectly modulate the physiological status of cashmere goats through the regulation of endogenous hormones and neurotransmitters, consequently mediating follicle developmental processes. The coordinated alterations in these pathways constitute the core transcriptional regulatory basis underlying CS-mediated cashmere growth promotion.

To further elucidate the regulatory logic of the “CS–sulfur nutrition–hair follicle development” axis, metabolomic profiling of skin tissues was performed. The results revealed that CS treatment-induced differential metabolites were predominantly enriched in pathways related to lipid metabolism, organic acid metabolism, and heterocyclic compound metabolism, with L-cysteine-glutathione disulfide and α-lipoic acid identified as two key sulfur-containing differential metabolites. CS can generate cysteine by cleaving disulfide bonds in cystine, providing a direct substrate for glutathione synthesis. Concurrently, its metabolite taurine can upregulate the activities of enzymes involved in glutathione metabolism, thereby enhancing systemic antioxidant capacity, alleviating oxidative damage to hair follicles, and sustaining normal follicular developmental cycles (35). Moreover, significant accumulations of amino acid metabolites including D-serine and L-threonine were observed, indicating that CS effectively supplements the organic sulfur pool, partially substituting for the functions of methionine and cystine, and participates in the remodeling of glycolipid and amino acid metabolism, thereby ensuring an adequate supply of substrates for keratin synthesis at the material level.

Integrating the transcriptomic and metabolomic datasets, the present study preliminarily elucidates the core regulatory framework through which CS modulates cashmere growth: dietary CS supplementation, on one hand, supplies an endogenous organic sulfur source, enriches sulfur-containing metabolic intermediates, and reshapes cutaneous amino acid and lipid metabolic homeostasis, thereby providing a critical material foundation for secondary follicle keratin synthesis and development; on the other hand, it orchestrates hair follicle development-related signaling pathways, including cAMP, bile acid, and thyroid hormone signaling, to activate hair follicle stem cell proliferation and differentiation, ultimately achieving enhanced cashmere growth and improved quality traits. Although the multi-omics integrative analysis has unveiled the regulatory potential of CS, the precise targeting mechanisms of the key sulfur-containing metabolites and functional genes, as well as the coordinated regulatory patterns of sulfur metabolic networks, warrant further validation. Future investigations will focus on functional characterization of follicle-specific key genes and sulfur-containing metabolites, in conjunction with serum GH and IGF-1 level measurements, to systematically refine the molecular mechanistic framework of CS-mediated sulfur nutrition in regulating cashmere traits.

## 5 Conclusion

This study explored the transcriptomic and metabolomic characteristics associated with the regulatory mechanism of cysteamine on cashmere production in cashmere goats, investigated the roles of DEGs and DEMs in the shared pathways including bile acid secretion pathway, cAMP signaling pathway, thyroid hormone synthesis, tryptophan metabolism pathway and renin secretion pathway after cysteamine supplementation, as well as the correlation between metabolites and genes. This study preliminarily suggests that cysteamine may be involved in the potential regulation of cashmere production traits in cashmere goats through the aforementioned pathways. Nevertheless, further functional verification studies are still required to systematically investigate the regulatory effects of cysteamine (CS) on sulfur-containing metabolites and pivotal regulatory genes within cashmere hair follicles, thereby providing more solid theoretical and scientific evidence for the dietary supplementation of CS in cashmere goats.

## Data Availability

Data are available via the SRA under accession number PRJNA1416633. Relevant metabolomic data have been deposited in OMIX under the project accession number OMIX017040.

## Footnotes

1 https://www.metaboanalyst.ca/.

2 https://magic-plus.novogene.com/.

## Supplementary material

SUPPLEMENTARY TABLE S1 Gene expression profile.

SUPPLEMENTARY TABLE S2 Differentially expressed genes (LCS VS CON).

SUPPLEMENTARY TABLE S3 Differentially expressed genes (HCS VS CON).

SUPPLEMENTARY TABLE S4 Differentially expressed genes (HCS VS LCS).

SUPPLEMENTARY TABLE S5 The KEGG enrichment information of DEGs.

SUPPLEMENTARY TABLE S6 740 Metabolites information overview table.

SUPPLEMENTARY TABLE S7 Cluster analysis of all differential metabolites.

SUPPLEMENTARY TABLE S8 Differential metabolites obtained among groups (CON, LCS, and HCS).
