# Supplementary material for: Deciphering cysteamine’s impact on cashmere-producing traits of cashmere goats: a transcriptomic and metabolomic perspective
Source: Front Vet Sci. 2026 Sep 17;13:1925080. doi: 10.3389/fvets.2026.1925080 (PMC13629525; doi:10.3389/fvets.2026.1925080)
Supplement: Supplementary file 1 [file Data_Sheet_1.docx]

Supplementary Material

# 1 Supplementary Figures and Tables

## 1.1 Supplementary Figures

**Supplementary Figure 1.** Venn of gene co expression.

**Supplementary Figure 2.** Correlation analysis of QC samples.

**Supplementary Figure 3.** Chart of OPLS-DA score and Response permutation testing.
